# Evaluation of Anticoagulation Practice With New-Onset Atrial Fibrillation in Patients with Sepsis and Septic Shock in Medical Intensive Care Unit: A Retrospective Observational Cohort Study

**DOI:** 10.7759/cureus.10026

**Published:** 2020-08-25

**Authors:** Karuppiah Arunachalam, Arvind Kalyan Sundaram, Kunal Jha, Lokendra Thakur, Kyle Pond

**Affiliations:** 1 Internal Medicine/Cardiology, Northeast Ohio Medical University, Canton, USA; 2 Critical Care, Geisinger Medical Center, Danville, USA; 3 Internal Medicine, Geisinger Medicine Center, Danville, USA; 4 Cardiology, Mount Auburn Hospital/Harvard Medical School, Cambridge, USA

**Keywords:** atrial fibrillation, critical care, anticoagulation, severe sepsis, septic shock

## Abstract

Objective

To investigate the anticoagulation practice in patients presenting with new-onset atrial fibrillation (NOAF) during sepsis and septic shock with one-year follow-up since discharge and to evaluate factors associated with the development of NOAF.

Methods

A retrospective observational cohort study was conducted using chart review in patients diagnosed with sepsis and septic shock.

Results

There was a total of 1132 patients diagnosed with sepsis and septic shock over a one-year period. Thirty-two patients were found to have NOAF in the setting of sepsis. Of this, eight (25%) patients were anticoagulated with warfarin and 14 (44%) patients were not anticoagulated during discharge. At one-year follow-up post-discharge, nine (29%) patients continued on warfarin and 16 (52%) patients remained not anticoagulated.

Conclusion

We found that the majority of patients who developed NOAF did not get anticoagulated at the time of discharge. A similar trend followed after one year of follow-up. Since proper treatment guidelines are not in place, these patients are at high risk for recurrent atrial fibrillation, stroke, transient ischemic attack, and death.

## Introduction

New-onset atrial fibrillation (NOAF) occurs in 4% to 20% of patients suffering from sepsis, 46% during septic shock, and 30% to 50% in post cardiothoracic surgery patients. Previous studies have shown that in patients with sepsis the onset of cardiac arrhythmias, particularly NOAF, correlates well with disease severity and is linked to morbidity rates, cardio-embolic events, heart failure, and extended hospitalization [1-3].

A number of risk factors have been identified for the development of NOAF, most notably heightened inflammatory state. Sepsis is characterized as a hyper-inflammatory condition involving the release of pro-inflammatory cytokines and interleukins. The induction of sepsis-induced atrial fibrillation (AF) has been attributed to the generation of an anatomical and electrophysiological substrate from severe inflammation [4,5]. Sepsis is a hyper-inflammatory state that leads to depressed myocardial function. Large volume fluid resuscitation causes an acute increase in left ventricular end-diastolic pressure and subsequent left atrial stretch. The combined mechanism provides an anatomical substrate due to which atrial fibrillation can occur in a septic patient [6].

Clinical practice guidelines indicate that the alleviation of underlying conditions such as sepsis ameliorates the risk of developing NOAF. The long-term effects of NOAF in sepsis patients remain unclear. This study aims to evaluate the factors that are associated with NOAF during sepsis, current practice regarding anticoagulation during hospital admission and on one-year follow-up.

## Materials and methods

The study was designed as a retrospective single-center, observational cohort study. It was performed at a tertiary care hospital with a 34-bed medical Intensive Care Unit (ICU) at Cambridge, USA. Our primary objective was to evaluate the anticoagulation practice during and after discharge in NOAF patients admitted with sepsis and septic shock. Secondary objectives were to determine the incidence, outcomes of NOAF and evaluate risk factors associated with NOAF. All consecutive adult patients age 18 years and older, with sepsis, severe sepsis, and septic shock admitted to the medical ICU from January 1, 2010, to December 30, 2013, were included. Patients with NOAF were considered the study group and patients without AF were included as the control group. Their medical records were reviewed and evidence of arrhythmias were collected from the EKG records. Arrhythmias extending greater than 30 seconds were evaluated by two independent cardiologists. The accompanying electrocardiograms with singular AF cases were separately assessed to confirm diagnosis. 

NOAF was diagnosed if the AF episode occurred during ICU management of sepsis syndrome provided there was no prior AF documentation in the patient’s electronic medical record. Exclusion criteria were patients with known history of intermittent AF, chronic heart failure, history of cardiac surgery, and patients admitted with ST-Elevation Myocardial Infarction (STEMI) and non-STEMI.

Data collection

Patient demographics, CHA_2_DS_2_-VASc score, medical co-morbidities, and echocardiographic data were acquired from electronic chart on admission. Baseline demographic and laboratory parameters included age, sex, race, past history of hypertension, diabetes mellitus, coronary artery disease, white blood cell (WBC) count, peak C-reactive protein (CRP) levels, presence of septic shock, presence of bacteremia, and source of infection, day of onset of atrial fibrillation after ICU admission, echocardiographic parameters, and treatment strategy were obtained. The data was recorded retrospectively one day before the onset of atrial fibrillation in the control group. Digitally stored echocardiogram results such as left ventricular ejection fraction, grade of mitral regurgitation, and left atrial size were obtained. Informed consent was waived because it was an observational retrospective study and the Institutional Review Board of the Mount Auburn Hospital approved this study (Protocol number- 032-2014). All patients who developed NOAF were followed up for a year to assess morbidity and mortality associated with NOAF after discharge from the hospital.

Statistical analysis

Statistical Product and Service Solutions (SPSS) statistical software, version 21, (IBM Corp., Armonk, NY) was used for statistical analyses. Baseline clinical characteristics and demographics were compared among patients with NOAF and the control group. Multivariate linear regression was performed to assess independent association of NOAF with continuous outcomes and chi-square test was done for categorical variables. All patients with NOAF were followed up for one year after admission to the ICU to assess treatment outcomes. P-value of less than 0.05 was considered to be significant for all analyses.

## Results

The total number of patients enrolled for the study was 1145 for a total of three years. NOAF was found in 32 patients with an incidence of 2.8%. A total of 88 patients were randomly chosen as control subjects. The mean age among 32 patients with new-onset atrial fibrillation was 76.2 years while the mean age among control subjects was 74.8 years.

There were a total of 15 (47%) male subjects in the NOAF group and 46 (52%) in the control group. Likewise, there were 17 (53%) female subjects in the NOAF group and 42 (48%) in the control group. The white population was predominant in both groups, 30 (94%) patients in NOAF and 81 (92%) in the control group. NOAF patients were predominantly hypertensive and non-diabetic. These comorbidities didn’t have any significant impact on the NOAF. Prevalence of coronary artery disease was lower both in the study and control group. NOAF patients also had significantly high septic shock during the sepsis episode (Table 1).

**Table 1:** Baseline characteristics of patients AF: Atrial Fibrillation; CRP: C-reactive protein; No AF: No Atrial Fibrillation (Control Group); CAD: Coronary Artery Disease

Variables	AF (N=32)	No AF (N=88)	Total (N=120)	p Value
Sex				0.6
Male	15 (47%)	46 (52%)	61 (51%)	
Female	17 (53%)	42 (48%)	59 (49%)	
Race				0.75
White	30 (94%)	81 (92%)	111 (92%)	
Others	2 (6%)	7 (8%)	9 (8%)	
History of Diabetes Mellitus				0.76
Yes	8 (25%)	20 (23%)	28 (23%)	
No	24 (75%)	68 (77%)	92 (77%)	
History of Hypertension				0.29
Yes	24 (75%)	57 (65%)	81 (68%)	
No	8 (25%)	31 (35%)	39 (32%)	
History of CAD				0.82
Yes	6 (19%)	15 (17%)	21 (18%)	
No	26 (81%)	73 (83%)	99 (82%)	
Septic Shock				0.03
Yes	6 (19%)	5 (6%)	11 (9%)	
No	26 (81%)	83 (94%)	119 (91%)	
Bacteremia				0.23
Yes	18 (56%)	60 (68%)	78 (65%)	
No	14 (44)	28 (32%)	32 (35%)	
Source of Infection				0.085
Lung	12(38%)	16(18%)	28(23%)	
Urinary Tract	13(40%)	45(51%)	58(48%)	
Others	7(22%)	27(31%)	34(29%)	
White Blood Cell Count				0.33
<3.5 K/cmm	0 (0%)	3 (3.5%)	3 (2.5%)	
3.5-11 K/cmm	7 (22%)	31 (35%)	38 (32%)	
11-20 K/cmm	16 (50%)	35 (40%)	51 (43%)	
>20 K/cmm	9 (28%)	19 (21.5%)	28 (22.5%)	
Peak CRP Levels				0.14
<300 mg/dl	22 (69%)	74 (84%)	96 (80%)	
≥300 mg/dl	10 (31%)	14 (16%)	24 (20%)	

Mostly atrial fibrillation occurred within 24 hours of admission, 21 (66 %) patients had AF in first 24 hours, two (6%) patients had AF in the first 48 hours, and seven (22%) patients had AF after 48 hours and two (6%) patients after 72 hours (Table 2). Among the study group, 30 (94%) patients had CHA2DS2-VASc score of 2 and more, one (3%) patient had score of 0 and 1 each (Table 2).

**Table 2:** Baseline Characteristic of patients with new-onset atrial fibrillation AF: atrial fibrillation

Variables	AF (N=32)
Time of onset of AF	
<24 hrs	21 (66%)
24-48 hrs	2 (6%)
48-72 hrs	7 (22%)
>72 hrs	2 (6%)
CHA_2_DS_2_-VASc Score	
0	1 (3%)
1	1 (3%)
≥2	30 (94%)
Echocardiographic Parameters	
Grade of Atrial Enlargement	
None	13 (41%)
Mild	13 (41%)
Moderate	4 (12%)
Severe	1 (3%)
Data Unavailable	1 (3%)
Grade of Mitral Regurgitation	
None	9 (28%)
Mild	17 (53%)
Moderate	4 (13%)
Severe	1 (3%)
Data Unavailable	1 (3%)
Ejection Fraction	
45-50%	5 (16%)
50-60%	26 (81%)
Data Unavailable	1 (3%)

Echocardiogram was not done in one out of 32 patients. On echocardiogram analysis of the study group, 13 (41%) patients didn’t have atrial enlargement, 13 (41%) patients had mild atrial enlargement, four (12%) patients had moderate atrial enlargement and one (3%) patient had severe atrial enlargement (Table 2). Likewise, echocardiogram analysis for grade of mitral regurgitation, nine (28%) patients didn’t have mitral regurgitation (MR), 17 (53%) patients had mild MR, four (13%) patients have moderate MR and one (3%) have severe MR (Table 2). Among the study group, ejection fraction (EF) of 55-60% was seen in 26 (81%) patients and EF of 45-50% was seen in five (16%) patients (Table 2).

**Figure 1:**
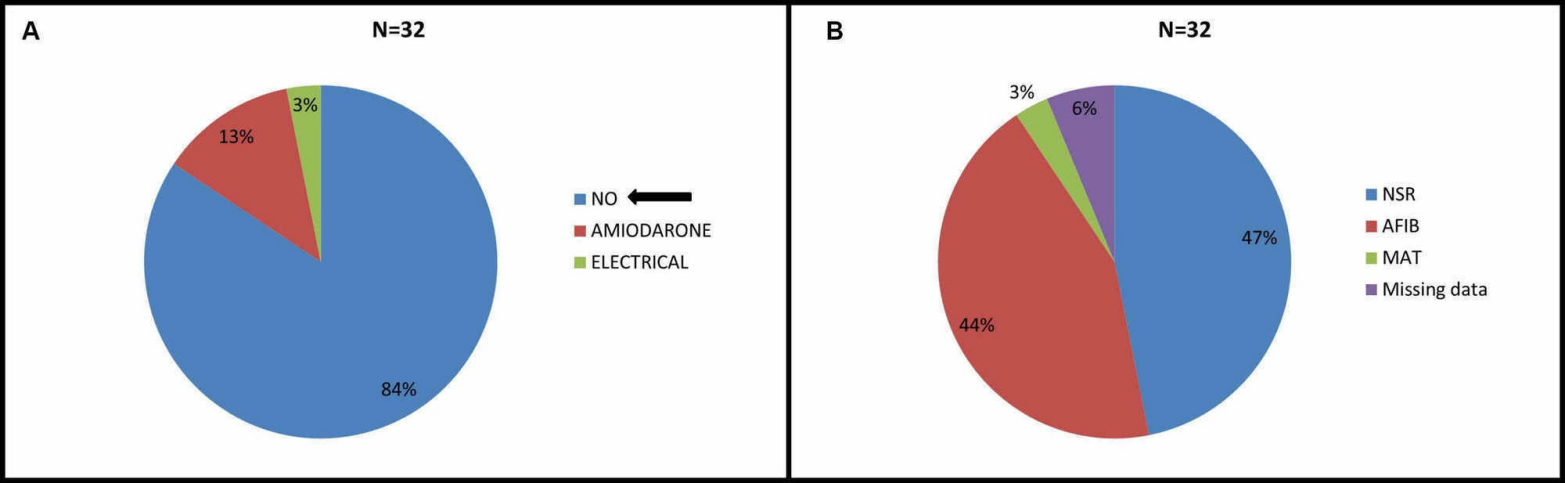
A: Management of Atrial Fibrillation B: Cardiac rhythm on discharge NSR- Normal sinus Rhythm, AFIB- Atrial Fibrillation, MAT- Multifocal Atrial Tachycardia

Out of 32 patients in study group, 27 (84%) were rate controlled with medications, four (14%) patients were given amiodarone and one (3%) patient was electrical cardioverted to sinus rhythm (Figure 1A).

At the time of discharge, sinus rhythm was restored in 15 (47%) patients, 14 (44%) patients remained in atrial fibrillation, two (6%) patients’ records couldn’t be obtained and one (3%) patient was found to have multifocal atrial tachycardia (Figure 1B).

**Figure 2:**
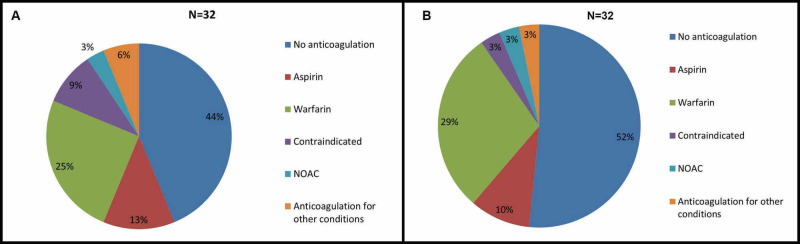
A- Anticoagulation strategies for new onset Atrial fibrillation during sepsis on discharge. B- One year followup of anticoagulation practice after new onset Atrial fibrillation after discharge NOAC- Newer Oral Anti-Coagulant.

On discharge, eight (25%) patients were discharged on warfarin, four (13%) patients were on aspirin, one (3%) patient was on newer oral anticoagulants, 14 (44%) patients didn’t receive anticoagulants, two (6%) patients received anticoagulants for other conditions like pulmonary embolism and deep vein thrombosis and anticoagulants were contraindicated in three (9%) patients (Figure 2A).

On one-year follow-up, nine (29%) patients were on warfarin, three (10%) patients were on aspirin, one (3%) patient was on newer oral anticoagulants, anticoagulant was contraindicated in one (3%) patient, one (3%) patient was anticoagulated for other conditions like pulmonary embolism, and 17 (52%) patients were not anticoagulated (Figure 2B).

## Discussion

Our study has shown that NOAF occurs in 2.8% of patients who presented with sepsis in the ICU, a frequency lower than that described in previous studies [7-9]. Chen et al. found that prevalence of NOAF was 7% within the sepsis patient population [10]. 

There was no association between increased white cell count and NOAF in patients suffering from sepsis in our research population. Mounting evidence has implicated systemic inflammation in the development of NOAF in critically ill patients, particularly those suffering septic shock. A number of studies have revealed a correlation between increased CRP levels and the onset and continuation of AF [11-13]. Indeed, CRP levels are increased two-fold in those with AF and additionally CRP levels were found to be higher in those suffering persistent AF than patients with paroxysmal AF, indicating the association between inflammation and the maintenance of AF [11]. The correlation between the inflammatory response and the onset of AF is further strengthened by our results that demonstrated increased CRP plasma concentration and WBC count during NOAF, however further prospective studies are required to validate this finding.

In our study, common triggers for AF such as age, hypertension, diabetes and heart disease did not affect the incidence of NOAF in septic patients, however, the occurrence of septic shock correlated well with NOAF (p<0.05) [14,15]. Walkey et al. showed that acute factors such as organ failure and nature of infection contributes to the development of NOAF, but this couldn’t be confirmed in our study [1,16,17]. We did not find any association between cardiovascular disease and NOAF in our study population. This could be secondary to small sample size and this finding needs to be evaluated in future [18,19].

Nearly 50% of the study population showed no evidence of atrial enlargement, and only a minority of patients had severe MR. Almost 81% of the patients had normal ejection fraction. Our results indicate that structural changes in the heart are not a causative factor for NOAF. Our study differs from some of the available literature that shows correlation of low EF with development of NOAF in sepsis patients [17,20].

In a recent analysis of 15,014 patients with critical illness in ICU, 1944 patients had sepsis and 1286 patients had septic shock. NOAF was not associated with increased mortality in critically ill patients in ICU but increased mortality seen among sepsis and septic shock patients with adjusted odds ratio of 1.24 (95% CI 1.1-1.39) and 1.28 (95% CI 1.14-1.44), respectively [21]. In another large study done in the Netherlands on 1782 patients, 418 patients were found to have 1087 episodes of NOAF and those patients were found to have increased mortality and longer stays in the hospital [22]. The cumulative risk of atrial fibrillation was 10% according to another study and the overall risk of mortality is very high in patients with NOAF during sepsis episode (hazard ratio (HR) 2.1, 95% CI 1.61-2.73). Length of stay and health care cost also increased in patients with NOAF during sepsis [22]. NOAF is associated with increased mortality in sepsis and septic shock patients on metanalysis [23]. Patients with NOAF during sepsis hospitalization were found to be older, have increased incidence of comorbid conditions such as congestive heart failure, myocardial infarction, increased length of stay and increased discharge health care cost [24].

Practice patterns of anticoagulation and their outcome was studied by Walkey et al. by analyzing 38582 patients with AF during sepsis. They reported that 35.3% of the patients were anticoagulated parenterally with intravenous heparin or subcutaneous enoxaparin with no indication for anticoagulation other than AF. Significant increase in bleeding risk without any difference in risk of ischemic stroke was revealed on parenteral anticoagulation. Before matched cohort analysis, there were 47520 patients, of which 8938 patients were on oral anticoagulation with warfarin in more than 80% of the patients. Oral anticoagulation was used in pre-existing AF compared to NOAF [25]. Additionally, in another single-center study of patients with preexisting AF during sepsis by Darwish et al., significant bleeding risk with parenteral anticoagulation during sepsis and ischemic stroke was not reported [26].

Most of these studies are limited by their retrospective design and uneven data collection. It is also known from previous studies that multiorgan dysfunction, coagulopathy and disseminated intravascular coagulation are common during severe sepsis and septic shock. Parenteral anticoagulation should be used with caution in patients with AF during sepsis episodes due to high risk of bleeding reported by those studies [27-29].

In our cohort, 94% had a CHA_2_DS_2_-VAS_C_ score of more than or equal to 2 but 44% of the patients were not anticoagulated due to bleeding risk or based on physician discretion based on clinical scenario. Present understanding is that AF is transient phenomenon in sepsis and resolves with clearance of sepsis . This leads to variation in anticoagulation practice by critical care physicians or cardiologists. We followed this cohort of patients for one year and found that 45% of patients in our study were continued on oral anticoagulation during outpatient follow-up as they developed persistent AF.

Contrary to the belief that NOAF is a transient acute phenomenon, this study reveals the long-term effects of NOAF during sepsis. It demonstrates that AF is not an acute issue that alleviates with sepsis management but can transform into persistent AF. Furthermore, the occurrence of NOAF during sepsis acts as an indicator for persistent AF and associated complications that should be taken into consideration during follow-up examination and medical care. The treatment of NOAF in patients with sepsis represents a major clinical challenge and currently there are no evidence-based strategies for prophylactic anticoagulant treatment. Pharmacological intervention may be beneficial to mitigate the risk of complications; however, methods must be implemented to sufficiently identify patients with the highest risk of developing NOAF during sepsis.

Walkey et al. in 2016 based on propensity-matched analysis of 39,693 patients with AF during sepsis found that calcium channel blockers are the most commonly used medication for NOAF during sepsis but beta blockers were shown to have superior outcomes. Digoxin and amiodarone therapy were used more in patients with heart failure and septic shock and were associated with increased hospital mortality [30].

Limitations

A number of limitations were encountered during the course of this study. First, retrospective observational studies are disposed to sampling errors and selection bias as the data is gathered from chart reviews. Second, the low sample size makes it difficult to prove conclusively a link between NOAF and morbidity rate in septic shock patients. Third, treatment of AF was not carried out by specific guidelines and as a result the inability to reestablish sinus rhythm must be considered carefully.

## Conclusions

We conclude that NOAF patients are not adequately anticoagulated so appropriate guidelines and management strategies need to be formulated for NOAF in sepsis. Appropriate anticoagulation therapy should be tailored for specific patient populations to mitigate stroke risk due to NOAF and proper caution measures are necessary to identify bleeding risks. It is highly essential to conduct a large randomized trial to assess stroke risk of NOAF and long-term consequences of NOAF during sepsis. Nevertheless, NOAF is associated with critically ill sepsis patients and leads to a complicated hospital course including longer length of stay and increased health care costs.
